# Bioactivity of Natural Compounds: From Plants to Humans

**DOI:** 10.3390/molecules31020295

**Published:** 2026-01-14

**Authors:** Guglielmina Froldi

**Affiliations:** Department of Pharmaceutical and Pharmacological Sciences, University of Padova, E. Meneghetti Building 2, 35131 Padova, Italy; g.froldi@unipd.it

Thanks to advancements in biochemistry, biology, and pathology, natural products (NPs) continue to be a subject of intense scientific investigation [1]. This ongoing interest is due to their rich composition of secondary bioactive metabolites, with valuable health properties. This Special Issue, entitled “Bioactivity of Natural Compounds: From Plants to Humans”, aims to explore this path further, investigating plant resources to discover potential new natural bioactives for improving health and treating human diseases.

A total of 17 papers have been published, comprising 14 research articles and 3 reviews. These studies range from the identification of bioactives from NPs to the investigation of their activities against pathogenetic cellular mechanisms. The phytoconstituents were studied as anti-inflammatory, antidiabetic, antiobesity, antibacterial, antiviral, and even potential anticancer agents. The experimental methods included in silico, in vitro, and in vivo investigations, all tools of preclinical drug development (Figure 1). Conservation of plant resources was also considered in this topic by studying NPs obtained from callus or, in general, plant cell cultures to reduce the use of wild or cultivated plants (contributions 1–3).

Among healthy plants and fruits, apples are a perfect example of a natural health resource. Apples (*Malus* × *domestica* (Suckow) Borkh., Rosaceae) are rich in various polyphenolic compounds, offering several health benefits to the human organism, primarily at the gastrointestinal level (contribution 4). The native active compounds (flavan-3-ols, phenolic acids, flavonols, dihydrochalcones, and anthocyanins) undergo significant metabolism by gastrointestinal and hepatic enzymes (first-pass metabolism), also producing new potential bioactives. Native polyphenols are more likely to be found at the gastric level and in the small intestine, while an increased amount of several secondary metabolites can be found in the colon and, after absorption and distribution, in the human organism. Among the beneficial effects, apple polyphenols have been shown to mitigate the adverse effects of nonsteroidal anti-inflammatory drugs on the gastrointestinal tract, potentially reducing the use of gastrointestinal protective drugs, such as proton pump inhibitors [2,3]. Moreover, apple polyphenols have been shown to control hyperglycemia in in vivo models and also in high–normal and borderline hyperglycemic human subjects [4].

Enhanced dietary intake of beans (*Phaseolus* spp., Fabaceae) is proposed to counteract inflammation, oxidative stress, and reduce the levels of cardiovascular and metabolic risk factors associated with lifestyle [5]. Bean bioactive compounds (polysaccharides, oligosaccharides, and polyphenols) decrease circulating inflammatory cytokines, downregulate their gene expression, and inhibit pathogen-associated pathways like the NF-κB pathway induced by LPS [6]. Beans also promote gut microbiota homeostasis, leading to increased short-chain fatty acid (SCFA) production and reduced pro-oxidant and inflammatory states (contribution 5). Their unique phytochemical profile induces nutrigenomic modifications that mitigate inflammation and oxidative stress. Anthocyanins and phenolic compounds in beans have a very low bioavailability (≤2%), also interfering with cytochrome activity. Furthermore, polyphenols inhibit key enzymes related to carbohydrate digestion, improve lipid profiles, and decrease inflammation markers and oxidative stress, suggesting their usefulness in preventing and treating metabolic diseases. The metabolites present in beans can qualitatively and quantitatively influence the intestinal bacterial flora, since beans are considered prebiotic foods that promote eubiosis by supporting a healthy gut microbiome [7]. Additionally, Adzuki bean (*Phaseolus angularis*), with its bioactive constituents, reduced body weight, regulated dyslipidemia, and improved glycemia [8].

*Alpinia oxyphylla* Miq. (Zingiberaceae), a well-known traditional Chinese medicinal plant, has been used to treat various neurological, urinary, and gastrointestinal ailments (contribution 6). In recent years, researchers have identified several new phytoconstituents, including terpenoids (eudesmane, oxyphyllol A and B, zingiberol), flavonoids (baicalein, wogonin, myricetin, and oxyphyllvonide derivatives), and various other compounds, such as diarylheptanoids, 20-propyl-β-sitosterol, and ferulic acid [9]. Pharmacokinetic studies have provided insights into the absorption, distribution, and metabolism of key compounds, showing that many bioactive compounds are distributed throughout the organism (rat) and metabolized mainly through glucoside conjugation, dehydration, desaturation, and glycine conjugation [10,11].

The study by Dalla Costa et al. (contribution 1) investigates the wound-healing and anti-inflammatory properties of *Sedum telephium* L. (Crassulaceae), a plant traditionally used in popular Italian medicine [12,13]. In vitro cultures of *S. telephium* were characterized and evaluated for their wound-healing activity using scratch assays on human skin cells, and for their anti-inflammatory potential on activated macrophages. The results demonstrated that extracts from these in vitro cultures promoted wound closure and exhibited significant anti-inflammatory effects. Building on this investigation, Dalla Costa et al. extended their research to *Morus alba* L. (Moraceae), exploring the potential of cell cultures as a sustainable source of anti-inflammatory compounds (contribution 2). The researchers optimized *M. alba* juices obtained from calli maintained under both photoperiod and dark conditions, harvested on the 14th and 28th days of the growth cycle, and from cell suspensions grown in dark conditions. They analyzed the phytochemical profiles of these juices with a focus on stilbenoids such as resveratrol derivatives. This research not only showcases the efficacy of *M. alba*-derived compounds but also highlights the advantages of plant cell culture technology in producing consistent, high-quality bioactive substances.

Anticancer drugs that efficiently counteract tumor growth are of high interest [14]. New treatments for triple-negative breast cancer (TNBC) targeting glutaminase 1 (GLS1), a key enzyme in cancer cell metabolism, are an important aim. Starting from ergosterol peroxide, a natural compound extracted from *Ganoderma lucidum* (Curtis ex Fr.) P. Karst. (Ganodermataceae), a series of new derivatives were obtained (contribution 7). Through structure–activity relationship studies and molecular docking, a new compound (**3g**, ergosterol peroxide-3-(5-((4-(dimethylamino)phenyl)amino)-5-oxopentanoate)) was found to be a promising GLS1 inhibitor. This compound showed significant anticancer effects in TNBC cell lines and xenograft models, reducing tumor growth and metastasis, suggesting ergosterol peroxide derivatives as a new class of GLS1 inhibitors for TNBC treatment. The study by Hermosaningtyas et al. investigated the potential anticancer properties of cell biomass derived from *Eryngium planum* L. (Apiaceae) and *Lychnis flos-cuculi* L. (Caryophyllaceae) callus against melanoma cells (contribution 3). Both callus extracts revealed the presence of phenolic acids and saponins, showing high cytotoxicity against melanoma cells, in a concentration- and time-dependent manner. These findings suggest that cell cultures of *E. planum* and *L. flos-cuculi* could be promising sources of compounds for melanoma treatment, offering a sustainable and standardized method for producing therapeutic NPs.

Recent studies have explored the potential of lapachol, found in *Tabebuia avellanedae* Lorentz ex Griseb. (Bignoniaceae) [15], in antimicrobial photodynamic inactivation (aPDI), which is an emerging approach to combat microbial infections. This naturally occurring naphthoquinone acts as a photosensitizer. When exposed to light of a specific wavelength, lapachol generates reactive oxygen species (ROS), which can cause oxidative damage to microbial cells. This photodynamic effect leads to the inactivation of various microorganisms, including bacteria and fungi. The compound showed significant efficacy against Gram-positive bacteria, completely inhibiting *Staphylococcus aureus* growth at 25 μg/mL, under blue light irradiation of 100 J·cm^−2^ (contribution 8). Scanning electron microscopy confirmed that lapachol-induced aPDI causes considerable bacterial cell wall damage, leading to cell lysis. This research provides insight into the potential use of lapachol as a natural photosensitizer in antimicrobial photodynamic therapy, addressing an urgent need for the search for new antimicrobial agents. Furthermore, in antiviral research, tigliane diterpenes from *Euphorbia nicaeensis* All. (Euphorbiaceae) demonstrate potent anti-HIV activity, highlighting the potential of natural products in fighting viral infections (contribution 9).

Tiliroside (kaempferol-3-O-(6″-p-coumaroyl)-glucoside), a flavonoid glycoside found in rose hips (*Rosa canina* L., Rosaceae), berries (e.g., *Fragaria* × *ananassa* Duchesne ex Rozier, Rosaceae), and several other plant sources, demonstrates anti-hyperuricemic effects by suppressing hepatic uric acid production and modulating its renal reabsorption, as evidenced by the ability of tiliroside to lower plasma and hepatic uric acid levels (contribution 10). This effectiveness, if clinically confirmed, suggests tiliroside as a dietary flavonoid with multi-target effects, such as anti-hyperuricemic, antidiabetic, antiobesity, and anti-hyperlipidemic activities [16,17].

β-Caryophyllene and farnesol, two characteristic sesquiterpenes detected in *Pterodon emarginatus* seeds (Fabaceae), were evaluated for their antiradical activity and cytotoxicity against HT-29 cells (contribution 11). The study of *P. emarginatus* extracts revealed a rich composition of volatile components, primarily sesquiterpenes, including α-copaene, β-caryophyllene, germacrene, spathulenol, and farnesol. The methanol extract demonstrated the highest antiradical activity, with all extracts showing appreciable antioxidant properties in DPPH and ORAC assays. Cytotoxicity tests indicated that the extracts, as well as β-caryophyllene and farnesol, exhibited no significant toxicity except at high concentrations (≥50 μg/mL). In silico studies predicted good intestinal absorption for β-caryophyllene and farnesol, potential to cross the blood–brain barrier, and a short half-life.

Various phytoconstituents, including flavonoids such as gossypetin (*Hibiscus sabdariffa* L., Malvaceae) and phytocannabinoids (*Cannabis sativa* L., Cannabinaceae), show promise in managing diabetes mellitus and prediabetes by protecting pancreatic β-cells and improving glucose homeostasis (contributions 12–13). In particular, phytocannabinoids reduced high-glucose–high-lipid (HGHL)-induced INS-1-cell apoptosis, likely by decreasing thioredoxin-interacting protein levels, and restored impaired glucose stimulated insulin secretion in HGHL-induced cells [18]. *Leonurus cardiaca* L. (Lamiaceae) is a well-known medicinal plant used in Europe to treat tachyarrhythmia, heart failure, and other cardiac disorders [19,20]. Kuchta et al., investigating nineteen isolated constituents, identified several compounds with potential PPAR (Peroxisome Proliferator-Activated Receptor) agonistic activity (contribution 14). Notably, 7-chloro-6-desoxy-harpagide showed significant PPAR-δ activation. Additionally, rutin, chicoric acid, and the novel compound cardiaphenyloside A demonstrated PPAR-α agonistic activity. Thus, *Leonurus* extracts may have potential applications in metabolic syndrome. Differently, compounds like 3-hydroxy-β-ionone from *Moringa oleifera* Lam. (Moringaceae) leaves exhibit significant anti-inflammatory effects, decreasing transendothelial migration of monocytes in EA.hy926 endothelial cells (contribution 15). Beyond disease-specific applications, *Curcuma longa* L. (Zingiberaceae) shows the ability to enhance stress resistance and extend lifespan in model organisms, suggesting potential applications in healthy aging (contribution 16). A purified lectin with a molecular mass of 34.4 kDa was recognized and purified from *Amaranthus hypochondriacus* L. (Amaranthaceae), with potential uses in biomedicine and biotechnology (contribution 17).

These investigations provide insights into potential new treatments and enhance the understanding of pharmacodynamics and pharmacokinetics of numerous phytoconstituents. Indeed, the traditional knowledge investigated by modern scientific approaches can potentially shed light on new pathways involved in human diseases. However, despite the progress, for many NPs, few investigations in vivo are available, particularly in human subjects. Moreover, it is unclear if their constituents act primarily as native molecules or rather as secondary metabolites formed during metabolism phases. Comprehensive studies are undoubtedly still necessary on the biotransformation and bioavailability of NPs. Furthermore, the role of NPs in personalized medicine, considering individual genetic variations and their impact on effectiveness, presents an exciting advancement for new research. Additionally, exploring ways to effectively integrate plant-derived compounds with conventional medical treatments could lead to more inclusive therapeutic approaches.

In conclusion, this Special Issue underscores the enduring significance of NPs as vital resources for human health, highlighting their potential to address a wide range of medical challenges and showing the way for future innovations in natural compound research and drug development. Readers are invited to explore the diverse studies presented in this Special Issue, which collectively advances our understanding of NPs and their bioactivities, from their origins in plants to their potential applications in human health.

Future perspectives:

1. Comprehensive in vivo studies, particularly in human subjects, to better understand the efficacy and safety of NPs.
2. Detailed research on the biotransformation and bioavailability of NPs in the human body.
3. Exploration of NPs in personalized medicine, considering individual genetic variations.
4. Investigation of natural bioactives helping in drug-resistant diseases.
5. Integration of plant-derived compounds with conventional medical treatments.
6. Continued development of sustainable and standardized plant cell culture techniques for producing medicinal substances.

## Figures and Tables

**Figure 1:**
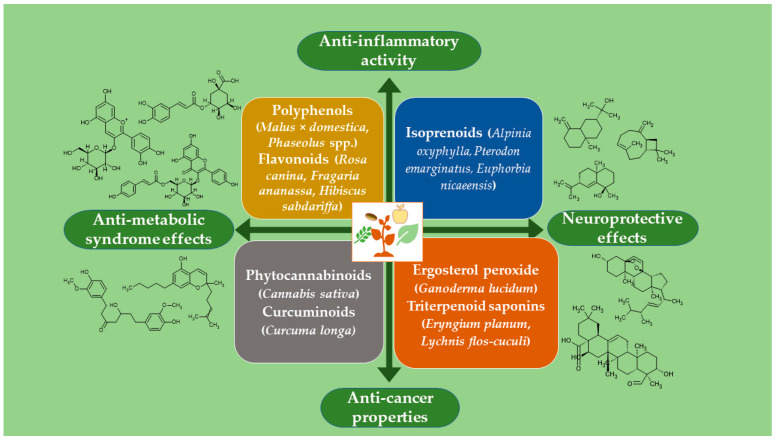
Plant-derived bioactive compounds and their potential health benefits. Chemical structures illustrate examples of polyphenols (cyanidin 3-O-glucoside, tiliroside, chlorogenic acid), isoprenoids (oxyphyllol A, zingiberol, β-caryophyllene), ergosterol peroxide, triterpenoid saponins (quillaic acid), phytocannabinoids (cannabichromene), and curcuminoids (hexahydrocurcumin) as discussed in the articles published in this book. Colored boxes indicate compound classes and plant sources. Green ovals show main health effects, as indicated by arrows.
